# Evaluation of Risk of Zoonotic Pathogen Transmission in a University-Based Animal Assisted Intervention (AAI) Program

**DOI:** 10.3389/fvets.2019.00167

**Published:** 2019-06-04

**Authors:** Sara F. Boyle, Virginia K. Corrigan, Virginia Buechner-Maxwell, Bess J. Pierce

**Affiliations:** ^1^Virginia-Maryland College of Veterinary Medicine, Virginia Polytechnic Institute and State University, Blacksburg, VA, United States; ^2^LMU College of Veterinary Medicine, Lincoln Memorial University, Harrogate, TN, United States

**Keywords:** animal assisted intervention, zoonoses, human-animal interactions (HAI), infection control, therapy animal

## Abstract

**Introduction:** Previous studies have shown that apparently healthy animals participating in Animal-Assisted Interventions (AAI) have the potential to asymptomatically carry and even transmit zoonotic pathogens to people, which is of particular concern for therapy animal teams visiting healthcare settings. This two-part study was designed to investigate the risk of zoonotic pathogen transmission within a university-based AAI program as a combination of the prevalence of these pathogens in the animal population as well as the handlers' understanding of the risks of zoonoses in AAI and their adherence to infection control practices.

**Methods:** In part one of the study, AAI program records were retrospectively reviewed and infectious disease screening test results were compiled from 22 dogs and 2 cats. Screening tests for dogs and cats included a zinc sulfate fecal float, fecal culture, and nasal and perianal skin swabs for methicillin-resistant *Staphylococcus aureus* (MRSA) and methicillin-resistant *Staphylococcus pseudointermedius* (MRSP). Additional tests for cats were blood cultures for *Bartonella henselae* and Toxoplasmosis IgG and IgM antibody titers. In part two, a survey was conducted of 40 registered therapy animal handlers to assess knowledge, attitudes, and perceptions regarding risk of infectious disease transmission in AAI settings, including risk to the animal, the handler, and those being visited.

**Results:** In part one, there were 17 total positive results of the 118 infectious disease screenings performed, 14 of which were potentially zoonotic organisms. In part two of the study, a majority (70%) of respondents expressed they had no concerns regarding infectious disease transmission in AAI settings. Despite handler education and guidelines, adherence to infection control practices was lacking.

**Discussion:** The results of this study support prior findings that animals participating in AAI can be asymptomatic carriers of zoonotic organisms. Compliance with infection control practices and hand hygiene are paramount to mitigate risk of zoonotic disease transmission, but was inconsistent among this group of handlers. Given the popularity of AAI programs in the U.S., similar studies should be performed on a larger scale to determine the level of adherence to currently recommended practices and potential need for improvement in infectious disease control education and/or policies.

## Introduction

Animal Assisted Interventions (AAIs) are commonplace in human healthcare facilities and other settings in the United States (1–3). Previous research published in the AAI literature suggest that apparently healthy animals participating in AAI programs have the potential to carry and spread zoonotic pathogens between animals and people (2). What is currently lacking in the AAI literature is an understanding of the human handler's knowledge of the risk of zoonotic disease transmission and adherence to infection control practices, which may be the biggest factor in reducing zoonotic transmission of infectious diseases in AAI situations.

Zoonotic pathogens can be spread via a multitude of routes including direct contact, such as bite wounds, scratches, licking, and petting; indirect contact with contaminated surfaces or bedding; as well as carrying vectors of diseases, such as fleas or ticks. Behavior evaluation and health screening can mitigate some of these risks, but handler education to ensure adherence to infection control policies is paramount in reducing the risk of transmission of pathogens, many of which can be carried asymptomatically by apparently healthy animals. In a study of therapy dogs in Ontario between May and July 2004, potentially zoonotic agents, including *Salmonella* spp., *Campylobacter* spp., *Malassezia pachydermatis, Clostridium difficile*, multi-drug resistant *Staphylococcus aureus* (MRSA), and others, were isolated from 80% of healthy dogs (4).

Within the realm of AAI, there is concern not only for zoonotic disease transmission from the animal to the people they visit, but also for the animals themselves and the handlers who may become infected by a pathogen their animal has transiently acquired while visiting. Dogs visiting healthcare facilities are at an increased risk of acquiring *C. difficile* and MRSA than those visiting other types of facilities, such as schools and group homes (5). It has also been demonstrated that AAI in long-term care facilities were associated with a greater probability of the animal acquiring a pathogen than in hospital settings, which was attributed to several factors, including varying infectious disease control and prevention practices as well as a higher percentage of elderly patients (5).

One report demonstrated that a therapy dog acquired MRSA from visiting a hospital (6). Another study demonstrated that a dog acquired *Clostridium difficile* while visiting an acute care facility (7). In addition, this study detected MRSA on the hands of one of the researchers after petting another dog that had just completed visiting a long-term care facility, which demonstrates that the transient contamination of a dog's fur can result in transmission to other humans, including their handler and others being visited (7). In each of these latter two cases, the dogs were reported to have engaged in behavior that increases the risk of disease transmission including “shaking paws,” in the case of the *C. difficile* dog, and repeatedly getting on beds and being kissed in the case of the MRSA dog (7).

A panel of experts recently examined the policies and procedures of several therapy animal organizations and facilities, as well as the available scientific literature, to develop a set of recommended guidelines for therapy animal organizations, facilities, and therapy animal handlers providing animal-assisted interactions in health care facilities (3). In this publication, the authors recommended that therapy animal organizations include training for handlers on zoonotic diseases and precautions that can be taken to mitigate the risk, particularly hand hygiene practices (3).

Hand hygiene is the most important infection prevention measure to reduce transmission of disease (3, 8–10). Hand sanitizer is a convenient alternative to hand washing, particularly in healthcare situations such as hospitals and nursing homes where patients may have mobility limitations that would make traditional handwashing more difficult. Hand sanitizer has been shown to be effective in reducing bacterial counts by up to 90% when used correctly in healthcare settings and still reduces bacterial counts significantly with less optimal use by >50% (11). While the effectiveness of hand sanitizer has not been specifically studied in therapy animal situations, it has been shown to be as effective as handwashing in agricultural settings such as livestock shows, petting zoos, and farm employees in reducing bacteria loads, particularly of enteric pathogens such as *Escherichia coli* and *Salmonella* (12, 13).

The recommendations from this paper also discuss reducing the risk of disease transmission by avoiding riskier behavior, such as taking treats and shaking paws. They also encourage bathing and grooming before visits, which reduce allergens and transient bacterial contamination (3, 10, 14).

The purpose of this two-part study was to evaluate the risk of zoonotic pathogen transmission in a university-based AAI program. The study involved examining not only at the prevalence of potentially zoonotic pathogens in the animal population involved in the program, but also the therapy animal handlers' perception of the risks of zoonoses and their self-reported actions taken that influence infectious disease transmission in AAI settings. The study hypothesis was that the majority of therapy animal handlers would report adherence to infectious disease control and prevention methods set forth by the affiliated National Therapy Animal Organization (NTAO).

## Methods

### Study Population

The study utilized information obtained from therapy animals and handlers from Virginia Tech Helping PAWS (Pet Assisted Wellness Services), an AAI program based out of the Virginia-Maryland College of Veterinary Medicine (VMCVM), between 2012 and 2017. VT Helping PAWS conducted its own training and testing of therapy animal teams until becoming an affiliate of a National Therapy Animal Organization (NTAO) in the fall of 2015. Handler education prior to this transition was addressed primarily through role-playing scenarios and emphasizing the importance of monitoring pets for signs of stress which could lead to an incident, and the recommendation to adhere to good hand-hygiene practices and to refrain from visits whenever they or their animal were ill. Discussions about zoonotic pathogens that could be spread during AAI were done individually by veterinary staff at the time of the animal's annual health screening.

Since the Fall of 2015, all VT Helping PAWS therapy animal teams are evaluated and subsequently registered by an NTAO, which offers a standardized curriculum for handlers that must be completed prior to the team evaluation with their animal. This course includes advocating for the animal's well-being to monitor for stress to prevent incidents, a strong emphasis on adherence to hand-hygiene, a brief overview of select zoonoses, and role-play scenarios to practice commonly encountered situations that may arise in AAI. Team evaluations must be renewed every 2 years for registration. Annual veterinary screenings are required to verify the animal is apparently healthy and free of infectious diseases, is up to date on their Rabies vaccination (with some species, such as rabbits, exempted), free of internal and external parasites, and not known to be fed a raw diet. The therapy animal teams visit a variety of AAI settings including college campus events, nursing home and assisted living facilities, libraries, school, hospitals, and more within the nearby community.

### Part 1: Infectious Disease Screening

As part of the certification process, prior to conducting AAI visits, dogs and cats were given annual physical exams, administered Rabies vaccination as required by law, prescribed flea, tick, and heartworm preventatives, and participated in standardized infectious disease screening according to protocols set forth by the college's biosecurity program between May 2012 and August 2015. Health screenings were repeated at least annually, and sooner if an animal had a positive result requiring treatment prior to resuming visits.

Program records were retrospectively reviewed and infectious disease screening test results were compiled. Screening tests for dogs and cats included a zinc sulfate fecal float for gastrointestinal parasites, fecal culture for pathogenic enteric bacteria, and nasal and perianal skin cultures for MRSA & MRSP. Cats were screened via blood cultures for *Bartonella henselae* and antibody titers for *Toxoplasma gondii*. At the time of screening, testing for *Clostridium difficile* was not routinely available as part of a fecal culture at VMCVM and therefore was not included in the screening process.

### Part 2: Handler Survey

The online survey was administered to the currently registered handlers belonging to VT Helping PAWS via email between April and May of 2017. This survey and its distribution were approved by the Virginia Tech Institutional Review Board (IRB #17-360). A copy of the survey questions can be found in the Supplementary Files.

The survey was designed to assess understanding of zoonoses and perceptions regarding risk-tasking and risk-reducing behaviors that affect infectious disease transmission in AAI settings. Risk-reducing behavior included using hand-sanitizer and use of a physical barrier when an animal sits on a person's lap. Risk-taking behavior included shaking/ giving paw, feeding treats, and not using hand-sanitizer or not washing their hands. Analysis was limited to descriptive statistics due to the small size of this pilot study.

## Results

### Infectious Disease Screening

Twenty dogs and 2 cats had infectious disease testing performed. A total of 118 tests were performed (104 for dogs, 14 for cats) and the results summarized in Table 1. There were 17 total positive results for dogs, 14 of which were potentially zoonotic organisms. Campylobacter was the most common zoonotic pathogen identified, representing nearly half (47%) of the 17 total positive results. Other potentially zoonotic pathogens identified included gastrointestinal parasites [*Giardia*, roundworms (*Toxocara canis*), and hookworms (*Ancylostoma caninum*)], and enteric bacteria species including enteropathogenic *Escherichia coli* (ETEC) and *Salmonella*. Dogs with gastrointestinal parasites were treated and had repeat fecal floats performed prior to conducting AAI visits. Dogs shedding pathogenic enteric bacteria were prescribed probiotics and advised to refrain from visitations with immunocompromised populations. Five of the six incidents of gastrointestinal parasites and 4 of the 11 cases of enteric bacteria were present at the first screening. Two dogs tested positive for both gastrointestinal parasites as well as pathogenic enteric bacteria. There were three dogs who tested positive for pathogenic enteric bacteria at least 2 years in a row, while four others tested positive only once. There were no positive results for cats. No dogs or cats cultured positive for MRSA/MRSP at any time point.

**Table 1:** Summary of infectious disease screening results from May 2012 to August 2015.

	**Zinc Sulfate Fecal Float**	**Fecal Culture**	**MRSA/MRSP Culture**	**Bartonella henselae culture (cats only)**	**Toxoplasma gondii serology (cats only)**
Positive results	6/41 (14.6%)	11/37 (29.7%)	0/ 34 (0%)	0/3 (0%)	0/3 (0%)
Species identified	*Isospora* (3), *Giardia* (1), *Ancylostoma caninum* (1), *Toxocara canis* (1)	Enteropathogenic *E. Coli* (2), *Campylobacter* (8), *Salmonella* (1)	N/A	N/A	N/A

### Survey Demographics

Responses were obtained from 20 of the 40 registered therapy animal handlers surveyed. Characteristics of the animals are summarized in Table 2. 19/20 (95%) of handlers were registered with one animal, while one handler was registered with two animals. Of these animals, 16 (76%) were dogs, two (10%) were cats, and three (14%) were other small animal species, which included one rat, one rabbit, and one bird. The average age of the animals was 4.5 years of age. The most common breed of dog was a mixed breed (5/16, 31%) followed by the Golden Retriever (3/16, 19%). Handler demographics were not obtained.

**Table 2:** Summary of therapy animal demographics.

	**Type of Pet**	**Number of Teams**	**Average Age**	**Age Range**
	Dog	16	4.9 years	1.5–11 years
	Cat	2	6 years	5–7 years
Other species	Bird	1	1.7 years	1–2 years
	Rabbit	1		
	Rat	1		

11/20 (55%) of the handlers had completed the NTAO's handler education course within the last 6 months, 4/20 (20%) had completed it within the last 6–12 months, 4/20 (20%) had completed it within the last 12–24 months, and only 1/20 (5%) had completed it more than 2 years prior. Sixty percent of the therapy animal handlers had completed their most recent team evaluation within the last 6 months, 4/20 (20%) completed it within the last 6–12 months and the remaining 4/20 (20%) had completed it within the last 12–24 months. Only two of the 20 handlers had completed an online course entitled “Infection Prevention and Control: Therapy Animal Visitation in Healthcare Settings,” which was based on the SHEA guidelines and designed to educate those involved with AAI about the risks of zoonoses (15).

**Figure 1:**
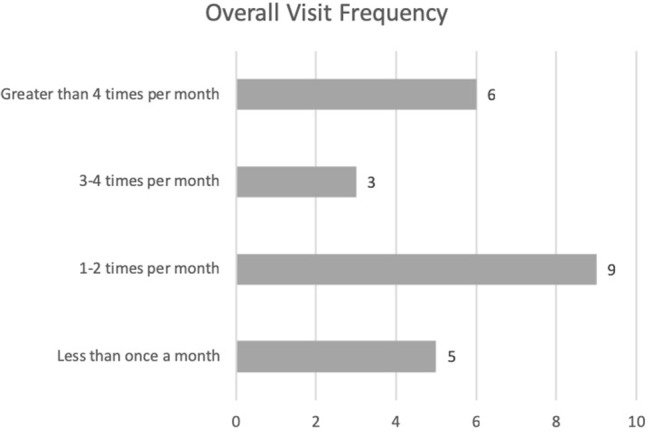
Overall visit frequency by therapy animal handlers.

**Figure 2:**
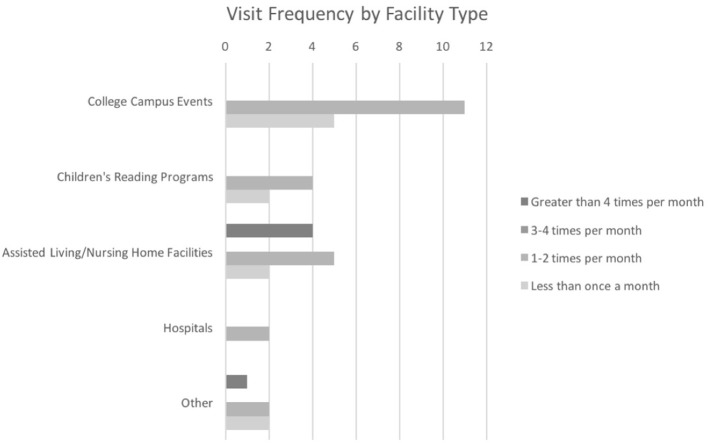
Visit frequency of therapy animal handlers by facility type.

Overall visit frequency is shown in Figure 1. 5/20 (25%) of the therapy animal handlers conducted AAI visits less than once per month, on average, while 9/20 (45%) visited one to two times per month. 3/20 (15%) of handlers visited an average of three to four times per month and the remaining 3/20 (15%) visited more than four times per month. A breakdown of the frequency of visits is shown in Figure 2. 16/20 (80%) of handlers participated in college campus events, 11/20 (55%) visited assisted living/nursing home facilities, 6/20 (30%) participated in children's reading programs, and 2/20 (10%) visited hospital patients. 4/20 (20%) of handlers reported that they visited other facilities including a children's museum, high schools, and a traumatic brain injury center. Most handlers (65%) visited more than one type of facility.

### Veterinarian Screening Requirements

A summary of the requirements that handlers reported their vet had in order to sign-off on the annual health screening form is shown in Table 3. Seven of the 19 (36%) of handlers reported that their veterinarian required a physical exam, fecal screening, heartworm testing and/or preventative, and flea/tick preventative. Only one handler reported that their veterinarian had specific recommendations for their animal's preventative care based on the fact they would be visiting as a therapy animal. This recommendation was to use oral flea/tick preventative instead of topical products due to increased bathing & grooming in preparation for AAI visits.

**Table 3:** Veterinary requirements to complete the annual health screening.

	**Number**	**Percentage (%)**
Physical exam	18/19	95
Fecal screening	13/19	68
Heartworm test and/or preventative	11/19	58
Flea/tick preventative	9/19	47

### Behaviors Influencing Risk of Infectious Disease Transmission

#### Bathing and Grooming

The top influential reasons why handlers chose not to bathe their animal before a visit are summarized in Table 4. Overall, 11/20 (55%) of therapy animal handlers expressed that visiting frequency influenced their decision of whether or not to bathe their animal in the 24 h preceding a visit. The animal's species influenced 3/20 (15%) of handlers, which were all handlers of species other than dogs. Animal preference was an influence for 4/20 (20%) of handlers. Other reasons were reported by 6/20 (30%) of handlers, with more than half of those indicating that time was a factor in their decision.

**Table 4:** Top influential reasons why animals are not bathed prior to AAI visits.

**Reason**	**Number**	**Percentage (%)**
Frequency of visits	11/20	55
Pet's preference	4/20	20
Time to bathe	4/20	20
Species of pet	3/20	15
Bathing frequency	1/20	5
Swimming	1/20	5
Health concerns	1/20	5

Of the 15 handlers with dogs, 10/15 (67%) expressed that visiting frequency influenced their decision to bathe their animal. One handler was influenced by their animal's preference, while 4/15 (27%) were influenced by other reasons. Of the five handlers who visit with cats and other species (excluding dogs), 3/5 (60%) were influenced by multiple reasons, all including animal's preference as a factor. 3/5 (60%) of the handlers indicated that species influenced their decision. Two handlers (40%) responded that other reasons, including time and health concerns, influenced their decision of whether or not to bathe their animal.

#### Hand Hygiene

**Figure 3:**
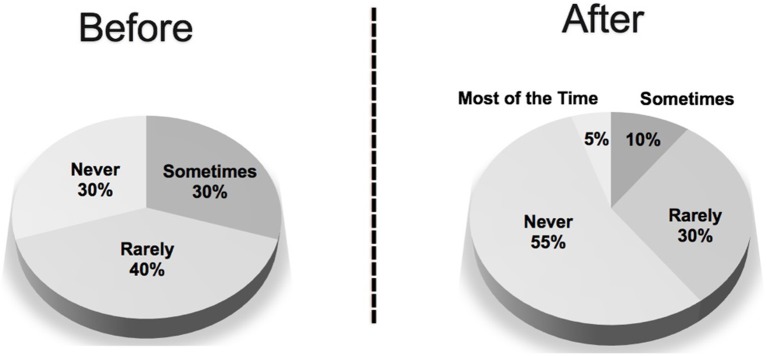
Hand sanitizer use by people visited by therapy animals before and after AAI visits as reported by therapy animal handlers.

A comparison of hand sanitizer use before and after visiting is shown in Figure 3. Hand sanitizer use was influenced by several factors. The largest influence was reported to be the person's willingness to use hand sanitizer, which was indicated by 13/20 (65%) of the handlers. 11/20 (55%) of handlers reported being influenced by the availability of hand sanitizer at the facility, and 30% reported being influenced by the facility type or facility policy. 6/20 (20%) reported they forget to offer it and 1/20 (5%) cited other reasons.

#### Risky Behaviors

**Figure 4:**
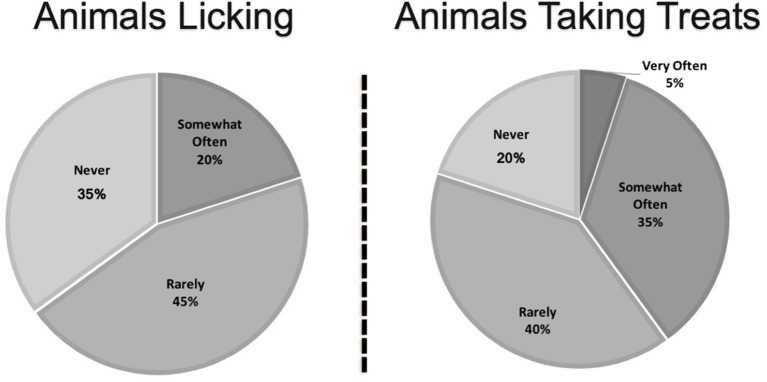
Frequency of animals engaging in high-risk behaviors as reported by therapy animal handlers.

**Figure 5:**
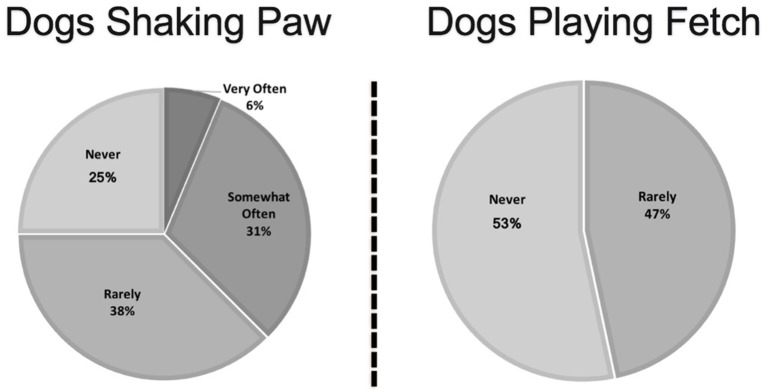
Frequency of dogs engaging in high-risk behaviors as reported by therapy animal handlers.

The frequency of the most common risky behaviors, including treat taking, licking, and shaking paw, are shown in Figures 4, 5. Of the six handlers who reported that their animal was on the bed in either a nursing home or a hospital, two reported they always used a barrier, two said they used a barrier most of the time, while one reported they rarely used a barrier, and one said they never used a barrier. Of the six small animals, four (66%) reported that their animal is held very often.

#### Risk Perception

When asked what diseases they were concerned about when visiting, 70% of handlers expressed that they had no concerns. Only 3/20 (15%) of handlers were able to name at least one zoonotic disease. Two of these three responses named *C difficile*. 3/20 (15%) of handlers said they had concerns, but were more general and did not state concern over a specific disease.

When asked if the risk of disease influenced their choice to visit, 65% of handlers responded that they were not influenced by the risk of disease. 4/20 (20%) said they might be influenced, while only 3/20 (15%) said they were influenced. Factors that would influence their choice to visit included sanitary conditions of the facility, staff hygiene behavior, and quarantine status of patients or facility. Two handlers stated that they suspend or limit their visits during flu season and one handler stated they simply do not visit medical facilities.

Handler concern of infectious disease transmission is summarized in Table 5. 7/20 (35%) of handlers were not at all concerned about the risk to themselves of acquiring a disease from the people they visit. 9/20 (45%) were somewhat concerned and 4/20 (20%) were moderately concerned. 10/20 (50%) of handlers were not at all concerned about the risk of their animal acquiring a disease from the people they visit. 6/20 (30%) were somewhat concerned, 3/20 (15%) were moderately concerned, and 1/20 (5%) were very concerned. 13/20 (65%) of handlers were not at all concerned about the risk of their animal transmitting a disease to the people they visit. 5/20 (25%) were somewhat concerned, 1/20 (5%) were moderately concerned, and 1/20 (5%) were very concerned. 15/20 (75%) of handlers were not at all concerned about the risk to their household of acquiring a disease from the people they visit. Of the three handlers who expressed they were moderately concerned about the risk to their household, two of the three live with someone who is immunocompromised, while the remaining handler indicated that their household make-up does influence choice of visit, but described concern for the other animals in their home rather than other people.

**Table 5:** Concern of disease transmission amongst therapy animal handlers.

	**Themselves Acquiring a Disease from Visiting**	**Animal Acquiring a Disease from Visiting**	**Animal Transmitting a Disease from Visiting**
Some Level of Concern	13/20 (65%)	10/20 (50%)	7/20 (35%)
No Concern	7/20 (35%)	10/20 (50%)	13/20 (65%)

**Figure 6:**
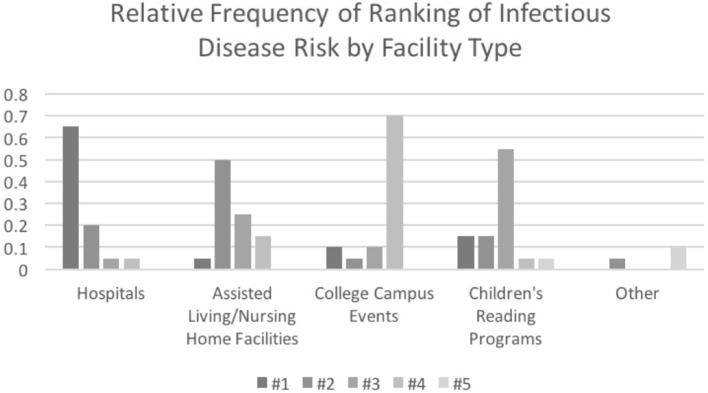
Perception of risk of infectious diseases by facility type as ranked by therapy animal handlers.

Handlers were asked to rank the infectious disease risk of hospitals, assisted living/nursing home facilities, children's reading programs, college campus events, and other types of events they attend, which is summarized in Figure 6. The majority (13/19) of handlers responding to this question ranked hospitals as the facility type with the highest risk for infectious diseases, while 21.1% ranked it as the second highest. Fifty two percent of handlers ranked assisted living/nursing home facilities as the second highest risk, while 26.3% felt it was the third most risky type of facility and 15.8% felt they were the least risky. 57.9% ranked children's reading programs as the third most risky, while 31.6% was split evenly between rating it as the riskiest or 2^nd^ most risky. Seventy three percent ranked college campus events as the least risky.

## Discussion

In the first part of this study, potentially zoonotic pathogens were found to be common in healthy, asymptomatic dogs and cats participating in a university-based AAI program, which is consistent with previous reports (4, 5, 7). Due to study limitations, it is not possible to draw further conclusions between facilities visited and subsequent positive results. The teams frequently visit a variety of places on a weekly to monthly basis while health screening was only performed annually. In addition, as many pathogenic enteric bacteria are transiently shed, the benefit of an annual culture is limited and is no longer recommended as part of health screenings for therapy animals.

In the second part of the study, important aspects of infection control were not consistently followed by therapy animal handlers despite the provided handler training provided by NTAO for the AAI program. In order to improve therapy handlers' knowledge of the risks of infectious disease transmission and improve their adherence to policies designed to mitigate the risk, a multidisciplinary approach is proposed, involving the therapy animal organization, the facilities being visited, and veterinarians involved with the AAI program.

### Role of Therapy Animal Organizations

Handler education needs to be assessed to determine why there is a lack of handlers' understanding of the potential risks of zoonoses to their animal and themselves when being involved in AAI. More handlers expressed concern about themselves acquiring a disease while visiting, such as the flu, than they were concerned about their animal transmitting or acquiring a disease. There simply was little concern despite the current education on infectious diseases provided by the NTAO. Despite (7) study demonstrating that visiting long-term health care facilities puts dogs at an increased risk of acquiring an infectious disease as compared to hospital visits, handlers predominantly perceived hospitals as riskier than nursing home type facilities. Only one handler felt it was the highest risk type of facility to visit.

There were insufficient responses available to be able to compare results between handlers who had taken the optional online module to those who had not, as only 2 handlers had taken the optional course. This would be an important consideration for future studies on this topic as the course is designed to increase handlers' knowledge and compliance of infection control practices. If this is in fact demonstrated, therapy animal organizations should ensure the information offered in the course is incorporated into the general education of all therapy animal handlers. However, it was noted in the survey that neither of the handlers that completed the optional course were able to name any diseases to be concerned about while visit despite the course specifically mentioning individual pathogens of concern.

Despite SHEA guidelines that recommend hand hygiene before and after each client, no handlers reported that people always used hand sanitizer before and after interacting with their animal (3). One possible reason for the non-compliance is that it isn't practical in certain situations such as schools and college visits where larger number of people are interacting with an animal at the same time. In larger group settings, ensuring hand hygiene becomes more difficult and handlers should be advised how to react in those situations to ensure they can mitigate the risk to their animal, themselves, and those around them.

Other reasons for non-compliance may be a lack of emphasis on hand hygiene as part of the team training, evaluation, and feedback process. If handlers are not expected to use hand hygiene during the evaluation, it may not become part of their routine. Handlers may need more guidance on how to handle the potentially uncomfortable questions that may arise from asking people to use hand sanitizer before interacting with the animal, as patients may feel that implies they are dirty or unclean.

Handlers may not recognize seemingly fun behaviors such taking treats, or even playing fetch as potential means of spreading zoonotic pathogens, so this needs to be presented as part of handler training. Pets' mouths have bacteria, so it is more commonly understood that licking could pose a risk, particularly to an immunocompromised person or if the person has a wound. However, it is also possible for the animal to pick up pathogens by licking, which could be passed to other people and/or animals it licks subsequently, or could be spread to its fur through grooming, which could then be picked up by another person when the animal is pet (5).

The SHEA guidelines do discourage treat giving unless there is a significant therapeutic benefit for a particular patient and that hand hygiene before and after is paramount to reducing the spread of pathogens (3). Treat giving is not just about the manner of taking the treat gently to avoid accidental bites, but a risky behavior that could allow for transmission of zoonotic pathogens, particularly bacteria. Handlers need to be educated that treat giving, playing fetch, and shaking paws are all risky behaviors, so that they are able to make educated decisions on whether or not to allow their animal to engage in these behaviors. They should also be provided information about how to reduce the risk of disease transmission if they choose to permit these activities.

Lastly, continuing handler education would also be beneficial. Currently, only the team evaluation is repeated every 2 years for continued registration, but handler education is conducted only once. Our study was too small to find significant differences in responses based on time since the handler course was completed, but this would be an important aspect to consider if this survey was conducted on a larger scale. Continued handler education is a way help handlers stay up to date on the latest research and recommendations.

### Role of the Facility

Facility policy and availability is a major influence on hand sanitizer use. It would be important to educate these facilities on the importance of hand hygiene with AAI. Facilities may be more focused on the potential benefits to their patients and not have access to the scientific literature that explains why hand hygiene during AAI is so important in reducing infectious disease transmission. It is a common fallacy that because these animals are apparently healthy, they are low risk for transmitting disease. In actuality, many can be asymptomatically carrying a variety of potentially harmful pathogens, which is particularly a concern for ill and/or immunocompromised patients.

As part of the SHEA guidelines to improve the safety of AAI visits in healthcare settings, education for facilities should also be developed to encourage adherence to the infection control practices to mitigate the risks to their patients and the teams that visit. The Pet Partners online course is also advertised as beneficial for healthcare facility employees (15). However, Pet Partners is but one of many therapy animal organizations and it is unknown how much marketing of this course is being conducted to reach facilities who participate in AAI so that visits can be made safer for all involved. One way of increasing awareness is to encourage therapy animal handlers to notify facilities they visit that the course is available. This would be particularly beneficial for places that do not have an infection control and prevention plan in place already.

### Role of the Veterinarian

Lastly, another aspect of education that could be improved is that of the veterinarians who provide medical care for the therapy animals. When assessing what veterinarians required to complete the health assessment form, not all veterinarians required a physical exam. This could be because the animal had one recently, which was not asked about in the survey. Only 68% required a fecal screening, but were still willing to sign off that the animal was free of internal parasites. What is more concerning is the lack of additional recommendations made when told the animal is going to be a therapy animal.

Educating veterinarians on the risks these animals are exposed to and the common ways that infectious diseases can be transmitted in AAI settings between animals and people may be a way to help close the knowledge gap. For example, therapy animals are bathed more frequently, so the suggestion of an oral vs. a topical flea/tick medication should be discussed. A veterinarian would be a good resource in choosing an appropriate cleansing shampoo or other grooming product to remove transient bacteria safely. They could help the handler choose a heartworm preventative that also routinely deworms, which may be a better choice for animals who are at higher risk of internal parasite transmission. Increased frequency of exams to twice a year just to be sure the animal is still physically sound and healthy has been recommended by several reports (1, 3).

### Role of the Therapy Animal Handler

The role of the therapy animal handler is perhaps the most important as they are the individuals choosing to take action to mitigate the risks of infectious disease transmission and make the visits as safe for all involved as possible.

The handlers generally reported adherence to the NTAO's policy of bathing 24 h before a visit. This policy is flexible in the case of frequent visiting or in certain species where bathing would actually be detrimental to the health of the animal. The handlers reported using a variety of other grooming methods as alternatives when bathing wasn't appropriate. However, handlers also frequently reported that time constraints also influenced their decision not to bathe. If an animal cannot be properly groomed before a visit, then the handler should decline visiting at that time and arrange for when they have sufficient time to prepare. With greater awareness of the SHEA guidelines, which recommend brushing before each visit and bathing when an animal is dirty or malodorous, handlers may be more able to comply with grooming recommendations as brushing is easier and quicker than bathing.

Of the handlers whose animals got in the beds of patients at healthcare facilities, only a small portion always used a barrier. One team reported they never used a barrier. In this study, the use of a barrier was required during the evaluation process of teams. However, in practice, the adherence was still low. Facility provided barriers may encourage greater use by handlers who can feel more confident that they are not bringing home a potentially contaminated blanket each time they visit.

With the guidance from NTAOs, their veterinarians, and the facilities they visit, handler adherence to these important infection control practices, particularly in health care settings should increase. Handler understanding and implementation of practices that reduce exposure is likely the best method for minimizing the risk zoonotic disease transmission during animal visitations.

### Study Limitations

This study is limited by the number of animals screened and the handlers responding to the survey. This survey was dependent on self-reported behavior as the researchers did not observe the teams' behavior on visits. This may lead to respondents overestimating perceived good behavior creating social desirability bias. However, the wording of the questions was designed to limit this as hanlders were only asked about the behaviors and the survey did not state which behaviors were considered risky vs. those that mitigated the risk. In addition, as the respondents' adherence to infection control practices was low, it is less likely that social desirability bias caused over inflation of these estimates.

Questions were not asked about hand washing, and therefore hand hygiene practices may have been somewhat underestimated. However, hand sanitizer use is typically more convenient and accessible in AAI situations so the impact of this omission was likely minimal.

As VT Helping PAWS is based out of a veterinary college, these results may not be generalizable to other communities of therapy animal teams. Handler education is somewhat variable as instructors may vary in their teaching methods and emphasis, while other handlers receive education via an online course. This is something that should be explored further in a larger survey.

The infectious disease screening and survey were conducted at different time periods by different investigators to minimize potential behavioral bias that screening results might provoke. Not all teams whose animals were screened were offered the opportunity to take the survey and vice versa.

At the time of the screening, the fecal culture performed did not include screening for *C. difficile*. As other studies have shown *C. difficile* as one of the more common pathogens found in therapy animal populations, positive test results were likely underestimated in our population.

## Conclusion

This is the first study to examine the adherence of therapy animal handers to infection control and prevention practices in an AAI program. Potentially zoonotic pathogens are commonly found on asymptomatic animals participating in AAI settings; however, routine zoonotic infectious disease screening of dogs and cats in AAI programs is not recommended (2, 3, 7). Strict adherence to infection control practices and hand hygiene are paramount, particularly for AAI in healthcare settings. It would be beneficial to conduct this study on a larger scale to determine if current handler education through therapy animal organizations are adequately preparing therapy animal handlers to respond to the potential risk of infectious diseases they may encounter while visiting in AAI settings. Additional emphasis on infection risk, control, and prevention should be provided as part of handler education as part of the process of registering a therapy animal team.

## Data Availability

All datasets generated for this study are included in the manuscript and/or the Supplementary Files.

## Ethics Statement

This study was carried out in accordance with the recommendations of Virginia Tech's Institutional Review Board(IRB) with written informed consent from all subjects. All subjects gave written informed consent in accordance with the Declaration of Helsinki. The protocol was approved by the IRB at Virginia Tech (IRB # 17-360).

## Author Contributions

VC conducted part 1 of study including retrospective data review and data analysis, supervised completion of part 2 of study, and completed revisions of article. SB conducted part 2 of study including study design, data collection and analysis, composed the original manuscript, and formatted manuscript based on feedback from other authors. VB-M assisted with multiple revisions of the manuscript in preparation for final publication. BP acquired data for part 1 of study and supervised completion of part 1. All authors have approved this manuscript for publication.

### Conflict of Interest Statement

The authors declare that the research was conducted in the absence of any commercial or financial relationships that could be construed as a potential conflict of interest.
