# Supplementary material for: Evaluation of Risk of Zoonotic Pathogen Transmission in a University-Based Animal Assisted Intervention (AAI) Program
Source: Front Vet Sci. 2019 Jun 4;6:167. doi: 10.3389/fvets.2019.00167 (PMC6558202; doi:10.3389/fvets.2019.00167)
Supplement: Supplementary file 1 [file Table_1.DOCX]

Survey Questionnaire

Q1 When did you initially complete the handler workshop?

- Within the last 6 months (1)
- 6 - 12 months ago (2)
- 12 - 24 months ago (3)
- Greater than 24 months ago (4)

Q2 How did you complete the handler workshop?

- Online (1)
- In-Person (2)

Q3 When did you initially pass your 1st evaluation?

- Within the last 6 months (1)
- 6 - 12 months ago (2)
- 12 - 24 months ago (3)
- Greater than 24 months ago (4)

Q4 When was your most recent evaluation?

- Within the last 6 months (1)
- 6 - 12 months ago (2)
- 12 - 24 months ago (3)
- Greater than 24 months ago (4)

Q5 Have you completed the Pet Partners Infectious Disease online module?

- Yes (1)
- In Progress (2)
- No (3)

Q6 How many therapy pets are you currently registered with?

- 1 (1)
- 2 (2)
- 3 (3)
- Other (please specify) (4) ____________________

Q7 What type of pet(s) do you visit with (check all that apply)?

- Small Dog (1)
- Medium-Large Dog (2)
- Cat (3)
- Bird (4)
- Rat (5)
- Guinea Pig (6)
- Rabbit (7)
- Equine (8)
- Mini Pig (9)
- Llama or Alpaca (10)
- Other (11) ____________________

*If “What type of pet(s) do you visit with (check all that apply)?”, Small Dog, Medium-Large Dog, Cat or Equine Is Selected:*

Q8a What breed is your pet?

*If “What type of pet(s) do you visit with (check all that apply)?”, Bird Is Selected*

Q8b What species of bird is your pet?

Q9a Age of 1st therapy pet (please specify age in months or years, ie. 8 months or 6 years)

Q9b Age of additional therapy pet(s) - (please specify ages in months or years, ie. 8 months or 6 years)

Q10 What types of visits do you participate in regularly with your therapy pet (check all that apply)?

- College Campus Events (1)
- Children's Reading Programs (2)
- Assisted Living/Nursing Home Facilities (3)
- Hospitals (4)
- Other (please specify) (5) ____________________

Q11 How often have you gone on visits as a therapy pet team, on average, over the last 6 months?

- Less than once a month (1)
- 1-2 times per month (2)
- 3-4 times per month (3)
- Greater than 4 times per month (4)

Q11a How often have you attended College Campus Events, on average, over the last 6 months?

- Less than once a month (1)
- 1-2 times per month (2)
- 3-4 times per month (3)
- Greater than 4 times per month (4)

*If “What types of visits do you participate in regularly with your therapy pet (check all that apply)?”, Children's Reading Programs Is Selected*

Q11b How often have you participated in Children's Reading Programs, on average, over the last 6 months?

- Less than once a month (1)
- 1-2 times per month (2)
- 3-4 times per month (3)
- Greater than 4 times per month (4)

*If “What types of visits do you participate in regularly with your therapy pet (check all that apply)?”, Assisted Living/Nursing Home Facilities Is Selected*

Q11c How often have you visited Assisted Living/Nursing Home Facilities, on average, over the last 6 months?

- Less than once a month (1)
- 1-2 times per month (2)
- 3-4 times per month (3)
- Greater than 4 times per month (4)

*If “What types of visits do you participate in regularly with your therapy pet (check all that apply)?”, Hospitals Is Selected*

Q11d How often have you visited Hospitals, on average, over the last 6 months?

- Less than once a month (1)
- 1-2 times per month (2)
- 3-4 times per month (3)
- Greater than 4 times per month (4)

*If “What types of visits do you participate in regularly with your therapy pet (check all that apply)?”, Other (please specify) Is Selected*

Q11e How often have you visited other locations, on average, over the last 6 months?

- Less than once a month (1)
- 1-2 times per month (2)
- 3-4 times per month (3)

Q12 At your pet's annual visit, what did your vet require to sign-off on the "Health Screening Form"?

- Physical Exam (1)
- Fecal Screening (2)
- Heartworm Test/Prescribe Heartworm Preventative (3)
- Flea/Tick Medication (4)
- Other (please specify) (5) ____________________

Q13 At your pet's annual visit, did your vet make any recommendations due to the fact your pet will be visiting as a therapy animal?

- Physical Exam (1)
- Fecal Screening (2)
- Heartworm Test/Prescribe Heartworm Preventative (3)
- Flea/Tick Medication (4)
- Other (please specify) (5) ____________________

Q14 How often is your pet bathed in the 24 hours before an event?

- Always (1)
- Most of the time (2)
- Some of the time (3)
- Rarely (4)
- Never (5)

Q15 What type of bathing products are you using? (check all that apply)

- Traditional shampoo/conditioner (1)
- Dry shampoo products (2)
- Grooming wipes (3)
- Other (please specify) (4) ____________________

Q16 What influences if your pet is bathed before a visit?

- Frequency of visits (1)
- Species of pet (2)
- Pet's preference (3)
- Other (please specify) (4) ____________________

Q17 How often do those petting your animal use hand sanitizer BEFORE touching your pet?

- Always (1)
- Most of the time (2)
- Some of the time (3)
- Rarely (4)
- Never (5)

Q18 How often do those petting your animal use hand sanitizer AFTER touching your pet?

- Always (1)
- Most of the time (2)
- Some of the time (3)
- Rarely (4)
- Never (5)

Q19 What influences whether someone uses hand sanitizer when interacting with your pet (check all that apply)?

- Availability at facility (1)
- Facility type/policy (2)
- Forget to offer (3)
- Person's willingness to use (4)
- Other (please explain) (5) ____________________

Q20 How often does your pet lick/give kisses on visits?

- Very often (1)
- Somewhat often (2)
- Rarely (3)
- Never (4)

*If “What type of pet(s) do you visit with (check all that apply)?”, Small Dog or Medium-Large Dog Is Selected*

Q21 How often does your pet shake/give paw on visits?

- Very often (1)
- Somewhat often (2)
- Rarely (3)
- Never (4)

*If “What type of pet(s) do you visit with (check all that apply)?”, Small Dog or Medium-Large Dog Is Selected*

Q22 How often does your pet play fetch/retrieve on visits?

- Very often (1)
- Somewhat often (2)
- Rarely (3)
- Never (4)

*If “What types of visits do you participate in regularly with your therapy pet (check all that apply)?”, Assisted Living/Nursing Home Facilities Is Selected*

Q23 How often does your pet get on people's beds when visiting assisted living/nursing home facilities?

- Very often (1)
- Somewhat often (2)
- Rarely (3)
- Never (4)

*If What types of visits do you participate in regularly with your therapy pet (check all that apply)? Hospitals Is Selected*

Q24 How often does your pet get on people's beds when visiting hospitals?

- Very often (1)
- Somewhat often (2)
- Rarely (3)
- Never (4)

*If “How often does your pet get on people's beds when visiting hospitals?”, Never Is Not Selected or If “How often does your pet get on people's beds when visiting assisted living/nursing home facilities?”, Never Is Not Selected*

Q25 How often do you use a barrier, such as a towel or blanket, when your pet get's on people's beds when they visit?

- Always (1)
- Most of the time (2)
- Some of the time (3)
- Rarely (4)
- Never (5)

*If “What type of pet(s) do you visit with (check all that apply)?”, Small Dog, Cat, Bird, Rat, Guinea Pig, or Rabbit Is Selected*

Q26 How often do other people hold your pet on a visit?

- Very often (1)
- Somewhat often (2)
- Rarely (3)
- Never (4)

If “How often do other people hold your pet on a visit?” Never Is Not Selected

Q27 How often do you use a barrier, such as a towel or blanket, when people hold your pet?

- Always (1)
- Most of the time (2)
- Some of the time (3)
- Rarely (4)
- Never (5)

Q28 What diseases are you concerned about when visiting?  If you are not concerned, please write 'none'.

Q29 What measures do you take to reduce infectious disease transmission between your pet and the people they visit?

- Bathing before visits (1)
- Hand Washing (2)
- Hand Sanitizer (3)
- Barrier (Blanket/Basket, etc.) (4)
- Other (please specify) (5) ____________________

Q30 Does the risk of infectious diseases influence your choice of facilities to visit?

- Yes (1)
- Maybe (2)
- No (3)

*If “Does the risk of infectious diseases influence your choice of facilities to visit?”, Yes or Maybe Is Selected*

Q31 If so, how?

Q32 Rank the following facility types based on their risk of infectious diseases (with 1 = highest risk)

______ Assisted Living/Nursing Home Facilities (1)

______ Hospitals (2)

______ College Campus Events (3)

______ Children's Reading Program (4)

______ Other (please specify): (5)

Q33 Please rate your level of concern with the following:

|  | Level of Concern | | | |
| --- | --- | --- | --- | --- |
|  | Not at all concerned (1) | Somewhat concerned (2) | Moderately concerned (3) | Very concerned (4) |
| The risk of your pet transmitting a disease to the people you and your pet visit? (1) |  |  |  |  |
| The risk of your pet acquiring a disease from the people you and your pet visit? (2) |  |  |  |  |
| The risk to yourself of acquiring a disease from the people you and your pet visit? (3) |  |  |  |  |
| The risk to those in your immediate household of acquiring a disease from the people you and your pet visit? (4) |  |  |  |  |

Q34 Is anyone in your immediate household immunocompromised?

- Yes (1)
- No (2)

Q35 Is anyone in your immediate household less than 5 years old?

- Yes (1)
- No (2)

Q36 Is anyone in your immediate household older than 65?

- Yes (1)
- No (2)

Q37 Does the make-up of your household influence your choice of facilities to visit?

- Yes (1)
- Maybe (2)
- No (3)

*If “Does the make-up of your household influence your choice of facilities to visit?”, Yes or Maybe Is Selected*

Q38 If so, how?
